# Supplementary material for: Footprint of Domestic Processing on Safety and Functional Properties of Italian Black Garlic
Source: Foods. 2025 Jul 24;14(15):2595. doi: 10.3390/foods14152595 (PMC12346592; doi:10.3390/foods14152595)
Supplement: Supplementary file 1 [file foods-14-02595-s001.zip › foods-3742774-supplementary.pdf]

Effect of process parameters on safety and functional aspects of black garlic from *Allium sativum* L.

Davide Addazii[1], Chiara Cevoli[1,2], Flavia Casciano[1], Federico Ferioli[1], Tullia Gallina Toschi[1,2], Andrea Gianotti[1,2,*], Lorenzo Nissen[1,2]

[1] DiSTAL - Department of Agricultural and Food Sciences, *Alma Mater Studiorum* – University of Bologna, Food Science Campus, P.za G. Goidanich, 60, 47521 Cesena, Italy 1; DA = davide.addazii2@unibo.it; CC = chiara.cevoli3@unibo.it; FC = cascianoflavia@yahoo.it; FF = federico.ferioli@unibo.it; TGT = tullia.gallinatoschi@unibo.it; AG = andrea.gianotti@unibo.it; LN = lorenzo.nissen@unibo.it

[2] CIRI - Interdepartmental Centre of Agri-Food Industrial Research, Alma Mater Studiorum – University of Bologna, P.za G. Goidanich, 60, 47521 Cesena, Italy e-mail@e-mail.com

* Correspondence: (LN) Lorenzo Nissen lorenzo.nissen@unibo.it ; (AG) Andrea Gianotti andrea.gianotti@unibo.it;

Table S1. Primers pairs employed for PCR and qPCR reactions and quantifications.

Table S2. ANOVA model for volatile compounds.

Table S3. ANOVA loadings for percentage of contribution of the volatilome.

Table S4. Supporting information for the Venn diagrams.

Table S5. Supporting information for the Pairwise intersection map.

Table S1. Primers pairs employed for PCR and qPCR reactions and quantifications.

Bacterial taxa	Target	Sequence 3′-5′	Bp*	MT**	Reference
Lactobacillales	*V3-V4*<br>*16S*	F-Lac: GCAGCAGTAGGGAATCT<br>R-Lac: GCATTYCACCGCTACACA	340	59.8<br>58.3	[45]
Bifidobacteriaceae	*RecA*	RecA-F: CGTYTCBCAGCCGGAYA<br>RecA-R: CCARVGCRCCGGTCATC	220	60.3<br>59.2	[46]
Escherichia coli	*FtsZ*	EcFtsZ-F: GGTATCCTGACCGTTGCT<br>EcFtsZ-R: ATACCTCGGCCCAGAACT	250	59.4<br>57.3	[47]

Table S2. ANOVA model for volatile compounds.

	Multiple	Multiple	Adjusted	SS	df	MS	SS	df	MS	F	p

Acetic acid	0.973793	0.948273	0.926104	1.422111	6	0.237019	0.077575	14	0.005541	42.7750	0.000000
Propanoic acid	0.820973	0.673996	0.534281	0.020337	6	0.003390	0.009837	14	0.000703	4.8241	0.007215
Formic acid	0.964965	0.931158	0.901655	0.012406	6	0.002068	0.000917	14	0.000066	31.5608	0.000000
Dodecanoic acid	0.941442	0.886313	0.837589	0.010355	6	0.001726	0.001328	14	0.000095	18.1908	0.000007
2-Furanmethanol	0.919164	0.844863	0.778376	0.003340	6	0.000557	0.000613	14	0.000044	12.7071	0.000058
Furfural	0.968211	0.937433	0.910618	0.870797	6	0.145133	0.058120	14	0.004151	34.9598	0.000000
5-Hydroxymethylfurfural	0.615733	0.379127	0.113038	0.002188	6	0.000365	0.003584	14	0.000256	1.4248	0.273085
2,5-Furandicarboxaldehyde	0.978625	0.957708	0.939582	0.000793	6	0.000132	0.000035	14	0.000003	52.8382	0.000000
2(5H)-Furanone	0.994839	0.989704	0.985292	0.008969	6	0.001495	0.000093	14	0.000007	224.3001	0.000000
Butyrolactone	0.988870	0.977864	0.968378	0.001957	6	0.000326	0.000044	14	0.000003	103.0770	0.000000
Hydroxy Acetone	0.943583	0.890348	0.843355	0.000248	6	0.000041	0.000031	14	0.000002	18.9462	0.000006
Pyrazine, methyl-	0.997974	0.995953	0.994218	0.178632	6	0.029772	0.000726	14	0.000052	574.1993	0.000000
Pyrazine, 2,6-dimethyl-	0.986268	0.972725	0.961036	0.017233	6	0.002872	0.000483	14	0.000035	83.2152	0.000000
Orcinol	0.837640	0.701642	0.573774	0.003284	6	0.000547	0.001397	14	0.000100	5.4872	0.004145
Phenol	0.713500	0.509082	0.298689	0.000517	6	0.000086	0.000499	14	0.000036	2.4197	0.081224
Nicotinyl alcohol	0.974584	0.949813	0.928305	0.000226	6	0.000038	0.000012	14	0.000001	44.1597	0.000000
1,3-Dithiane, 2-methyl-	0.971910	0.944609	0.920870	0.131160	6	0.021860	0.007691	14	0.000549	39.7916	0.000000
Butane, 1-isothiocyanato-	0.787839	0.620690	0.458128	0.000848	6	0.000141	0.000518	14	0.000037	3.8182	0.018224
1,3,5-Trithiane	0.899988	0.809979	0.728541	0.008532	6	0.001422	0.002002	14	0.000143	9.9460	0.000224
2,4-Dithiapentane	0.961078	0.923671	0.890958	0.001063	6	0.000177	0.000088	14	0.000006	28.2359	0.000000
1,3-Dithiane, 2,2-dimethyl-	0.982050	0.964422	0.949175	0.067802	6	0.011300	0.002501	14	0.000179	63.2508	0.000000
1,4-Dithiane	0.722671	0.522254	0.317506	0.040900	6	0.006817	0.037415	14	0.002672	2.5507	0.069828
1,3-Dithiane	0.942068	0.887493	0.839276	0.571443	6	0.095241	0.072441	14	0.005174	18.4061	0.000007
1,2-Dithiolane	0.884474	0.782295	0.688993	0.000249	6	0.000041	0.000069	14	0.000005	8.3845	0.000547
3-Vinyl-1,2-dithiacyclohex-4-ene	0.985574	0.971357	0.959082	0.050154	6	0.008359	0.001479	14	0.000106	79.1295	0.000000
Sulfide, allyl methyl	0.935108	0.874428	0.820611	0.110152	6	0.018359	0.015818	14	0.001130	16.2482	0.000014
Allyl mercaptan	0.978978	0.958398	0.940568	0.107262	6	0.017877	0.004656	14	0.000333	53.7536	0.000000
2-(Allylamino)-5-ethyl-1,3,4-thiadiazole	0.981259	0.962869	0.946955	0.357276	6	0.059546	0.013778	14	0.000984	60.5065	0.000000
Trisulfide, di-2-propenyl	0.949850	0.902214	0.860306	0.006986	6	0.001164	0.000757	14	0.000054	21.5284	0.000003
Diallyl disulphide DADS	0.968734	0.938445	0.912065	1.856122	6	0.309354	0.121747	14	0.008696	35.5733	0.000000
Diallyl sulfide DAS	0.959898	0.921405	0.887721	0.797285	6	0.132881	0.068008	14	0.004858	27.3546	0.000001
Dimethyl trisulfide DATS	0.991373	0.982821	0.975459	0.069319	6	0.011553	0.001212	14	0.000087	133.4939	0.000000
3-Thiopheneethanol	0.983626	0.967520	0.953600	0.000134	6	0.000022	0.000004	14	0.000000	69.5063	0.000000
1,3-Benzenedithiol	0.985727	0.971658	0.959511	0.000023	6	0.000004	0.000001	14	0.000000	79.9930	0.000000
Thiazole, 5-methoxy-	0.990453	0.980996	0.972852	0.000519	6	0.000086	0.000010	14	0.000001	120.4509	0.000000
2-Thiazolamine, 5-nitro-	0.928266	0.861678	0.802397	0.001395	6	0.000233	0.000224	14	0.000016	14.5355	0.000027

Table S3. ANOVA loadings for percentage of contribution of the volatilome.

VOC ID	ABt12	ASt12
Acetic acid	61.5	38.5
Propanoic acid	46.7	53.3
Formic acid	32.9	67.1
Dodecanoic acid	46.5	53.5
2-Furanmethanol	50.8	49.2
Furfural	62.8	37.2

5-Hydroxymethylfurfural	44.6	55.4
2,5-Furandicarboxaldehyde	0.0	100.0
2(5H)-Furanone	63.8	36.2
Butyrolactone	53.9	46.1
Hydroxy Acetone	71.4	28.6
Pyrazine, methyl-	100.0	0.0
Pyrazine, 2,6-dimethyl-	100.0	0.0
Orcinol	100.0	0.0
Phenol	34.7	65.3
Nicotinyl alcohol	0.0	100.0
1,3-Dithiane, 2-methyl-	75.0	25.0
1,3,5-Trithiane	100.0	0.0
2,4-Dithiapentane	0.0	100.0
1,3-Dithiane, 2,2-dimethyl-	39.0	61.0
1,4-Dithiane	33.5	66.5
1,2-Dithiolane	100.0	0.0
3-Vinyl-1,2-dithiacyclohex-4-ene	56.8	43.2
Sulfide, allyl methyl	55.1	44.9
DADS	35.7	64.3
DAS	52.6	47.4
DATS	45.1	54.9

Table S4. Supporting information for the Venn diagrams.

ABT0 ∩ ABT6 ∩ ABT12	ABT0 – (ABT6 ∪ ABT12)	ABT6 – (ABT0 ∪ ABT12)	ABT12 – (ABT0 ∪ ABT6)
1 3 5-Trithiane	Ethyl 1 3-dithiane-2-carboxylate	1-Propyne 1-(ethenylthio)-	Formic acid
1 3-Dithiane	Naphthalene 2-methyl-	4H-Pyran-4-one 3 5-dihydroxy-2-methyl-	1 3-Dithiane 2 2-dimethyl-
2-Pentanol 4-methyl-	Quinoline 2-neopentyl-	Oxypurinol (1H-Pyrazolo dione)	2(5H)-Furanone
3-Vinyl-1 2-dithiacyclohex-5-ene	1 3-Benzenedithiol	Maltol	Methyl 2-furoate
Butane 1-isothiocyanato-	Dimethyl-(6-methyl-2-thioxo-[1 3 2]oxathiaphosphinan-2-yl)-amine	Tetrahydrofuran 2-ethyl-5-methyl-	Propanoic acid
Butylated Hydroxytoluene	2-Thiouracil	3-Hydroxybiphenyl	2-Methyl-3(2-furyl)acrolein
Cyclohexasiloxane dodecamethyl-	2-Thiapentane 4-(9-borabicyclo[3.3.1]non-9-yloxy)-	Mequinol	Orcinol
Diallyl disulphide	Thiazole tetrahydro-	Allyl mercaptan	Eicosane
Diallyl sulfide	Dimethoate	2 5-Furandicarboxaldehyde	Butyrolactone
Dodecane	3-Thiopheneethanol	5-Hydroxymethyldihydrofuran-2-one	1 5-Naphthyridin-4-ol
Dodecanoic acid	2 6-Difluoropyridine	Estra-1 3 5(10)-trien-17-one 6-methoxy-3-[(trimethylsilyl)oxy]- O-methyloxime (6.beta.)-	Nicotinyl alcohol
Indole	1-Butene 1-(methylthio)- (E)-	(+)Borneol	
Phenol 2 4-bis(1 1-dimethylethyl)-	3-Vinyl-1 2-dithiacyclohex-4-ene	Pyrazine methyl-	
Phenol 4-(1 1 3 3-tetramethylbutyl)-	2-Vinyl-1 3-dithiane	Fumaronitrile	
Tetradecane	3H-1 2 4-Triazole-3-thione 2 4-dihydro-4-methyl-	Sulfurous acid hexyl 2-pentyl ester	
Trisulfide di-2-propenyl	Tetrasulfide di-2-propenyl	1-Propene 1-(methylthio)- (E)-	
	Hydrocoumarin	Thiophene 3-ethyl-	
	2-Hydroxy-3-methoxy-succinic acid dimethyl ester	Disulfide dimethyl	

	Dinocap	2-Propen-1-ol	
	Dibutyl 3 6 9 12-tetraoxatetradecane-1 14-dioate	Formic acid 2-propenyl ester	
	Methanethioamide N N-dimethyl-	Sulfide allyl methyl	
	1.2-diacetylhydrazine	Thiazole 5-methyl-	
	N-Carbethoxy-N-methoxyamine	dl-Alanyl-l-alanine	
	2-(Allylamino)-5-ethyl-1 3 4-thiadiazole	2(5H)-Furanone 5-methyl-	
**AST0 ∩ AST6 ∩ AST12**	**AST0 – (AST6 ∪ AST12)**	**AST6 – (AST0 ∪ AST12)**	**AST12 – (AST0 ∪ AST6)**
1 3-Dithiane	Methyl 2 3-anhydro-.beta.-d-ribofuranoside	Butanoic acid 3-methyl-	Ethanone 1-(1H-pyrrol-2-yl)-
2 4-Dithiapentane	Phenol 4-(1 1 3 3-tetramethylbutyl)-	Fumaronitrile	Butyrolactone
3-Vinyl-1 2-dithiacyclohex-5-ene	Thiazole 5-methoxy-	Dibutyl 3 6 9 12-tetraoxatetradecane-1 14-dioate	3-Hydroxybiphenyl
4-Thiazolidinone 3-amino-2-thioxo-	3-Thiopheneethanol	Orcinol	Pentacosane
Butylated Hydroxytoluene	2 6-Difluoropyridine	Allopurinol (4-Hydroxy pyrazolo)	Benzene 1 3-bis(1 1-dimethylethyl)-
Diallyl disulphide	Hexadecane	1-Oxa-4 6-diazacyclooctane-5-thione	Nicotinyl alcohol
Diallyl sulfide	2-Vinyl-1 3-dithiane	2-Propen-1-ol	
Dimethyl trisulfide	1.2-diacetylhydrazine	2-Propenoic acid 2-methyl-	
Dodecanoic acid	Disulfide dimethyl	Sulfurous acid hexyl 2-pentyl ester	
Indole	Hydrocoumarin	Tetracosane	
Phenol 2 4-bis(1 1-dimethylethyl)-	2-Hydroxy-3-methoxy-succinic acid dimethyl ester	Furan 2-ethyltetrahydro-5-methoxy-2-methyl-	
Propanoic acid	Allyl mercaptan	2 5-Furandione 3 4-dimethyl-	
Tetradecane	2-(Allylamino)-5-ethyl-1 3 4-thiadiazole	Octanoic acid 2-phenylethyl ester	
Trisulfide di-2-propenyl		Formic acid 2-propenyl ester	
		4H-Pyran-4-one 3 5-dihydroxy-2-methyl-	
		5-Hydroxymethyldihydrofuran-2-one	
		Pyrimidine 4-methyl-2-phenyl-	
		Oxypurinol (1H-Pyrazolo dione)	
		Pyrazine 2 6-dimethyl-	
		3 4-Dihydroxyproline	
		Benzaldehyde 2-fluoro-3-hydroxy-	
		Dehydromevalonic lactone	
		1-Propyne 1-(ethenylthio)-	
		1H-Purine 8-methyl-6-(methylthio)-	
		5-Acetoxymethyl-2-furaldehyde	
**AST0 ∩ ABT0 ∩ ABT12 ∩ AST12**	**(ABT12 ∩ AST12) – (AST0 ∪ ABT0)**	**(AST0 ∩ ABT0) – (ABT12 ∪ AST12)**	
1 3-Dithiane	1 3-Dithiane 2 2-dimethyl-	1 3-Benzenedithiol	
3-Vinyl-1 2-dithiacyclohex-5-ene	1H-Pyrrole-2-carboxaldehyde	1 3-Dithiane 2-methyl-	
Butylated Hydroxytoluene	2 5-Thiophenedicarboxaldehyde	1.2-diacetylhydrazine	
Diallyl disulphide	2(5H)-Furanone	2 6-Difluoropyridine	
Diallyl sulfide	2-Furancarboxaldehyde 5-methyl-	2-(Allylamino)-5-ethyl-1 3 4-thiadiazole	
Dodecanoic acid	2-Furanmethanol	2-Hydroxy-3-methoxy-succinic acid dimethyl ester	
Indole	2-Propanone 1-hydroxy-	2-Thiazolamine 5-nitro-	
Phenol 2 4-bis(1 1-dimethylethyl)-	4-Cyclopentene-1 3-dione	2-Vinyl-1 3-dithiane	

Tetradecane	4H-Pyran-4-one 2 3-dihydro-3 5-dihydroxy-6-methyl-	3-Thiopheneethanol	
Trisulfide di-2-propenyl	5-Hydroxymethylfurfural	3-Vinyl-1 2-dithiacyclohex-4-ene	
	Acetic acid	3H-1 2 4-Triazole-3-thione 2 4-dihydro-4-methyl-	
	Benzene 1 3-bis(1 1-dimethylethyl)-	5-(2-Thienyl)pentanoic acid	
	Butyrolactone	Amidinothiourea	
	Ethanone 1-(1H-pyrrol-2-yl)-	Hexadecane	
	Ethanone 1-(2-furanyl)-	Hydrocoumarin	
	Formic acid	Thiazole 5-methoxy-	
	Furfural		
	Methyl 2-furoate		
	Nicotinyl alcohol		
	Phenol		

Table S5. Supporting information for the Pairwise intersection map.

**Item**	**Occurrence**	**Present in**
**AB + AS**		
1 3-Dithiane	4	AST0, ABT0, ABT12, AST12
3-Vinyl-1 2-dithiacyclohex-5-ene	4	AST0, ABT0, ABT12, AST12
Butylated Hydroxytoluene	4	AST0, ABT0, ABT12, AST12
Diallyl disulphide	4	AST0, ABT0, ABT12, AST12
Diallyl sulfide	4	AST0, ABT0, ABT12, AST12
Dodecanoic acid	4	AST0, ABT0, ABT12, AST12
Indole	4	AST0, ABT0, ABT12, AST12
Phenol 2 4-bis(1 1-dimethylethyl)-	4	AST0, ABT0, ABT12, AST12
Tetradecane	4	AST0, ABT0, ABT12, AST12
Trisulfide di-2-propenyl	4	AST0, ABT0, ABT12, AST12
1 3 5-Trithiane	3	AST0, ABT0, ABT12
2 4-Dithiapentane	3	AST0, ABT0, AST12
4-Thiazolidinone 3-amino-2-thioxo-	3	AST0, ABT0, AST12
Butane 1-isothiocyanato-	3	ABT0, ABT12, AST12
Carbon dioxide	3	ABT0, ABT12, AST12
Cyclohexasiloxane dodecamethyl-	3	ABT0, ABT12, AST12
Dimethyl trisulfide	3	AST0, ABT12, AST12
Dodecane	3	ABT0, ABT12, AST12
Phenol 4-(1 1 3 3-tetramethylbutyl)-	3	AST0, ABT0, ABT12
Propanoic acid	3	AST0, ABT12, AST12
1 3-Benzenedithiol	2	AST0, ABT0
1 3-Dithiane 2 2-dimethyl-	2	ABT12, AST12
1 3-Dithiane 2-methyl-	2	AST0, ABT0
1.2-diacetylhydrazine	2	AST0, ABT0
1H-Pyrrole-2-carboxaldehyde	2	ABT12, AST12
2 5-Thiophenedicarboxaldehyde	2	ABT12, AST12
2 6-Difluoropyridine	2	AST0, ABT0
2(5H)-Furanone	2	ABT12, AST12
2-(Allylamino)-5-ethyl-1 3 4-thiadiazole	2	AST0, ABT0
2-Furancarboxaldehyde 5-methyl-	2	ABT12, AST12
2-Furanmethanol	2	ABT12, AST12
2-Hydroxy-3-methoxy-succinic acid dimethyl ester	2	AST0, ABT0
2-Propanone 1-hydroxy-	2	ABT12, AST12
2-Thiazolamine 5-nitro-	2	AST0, ABT0
2-Vinyl-1 3-dithiane	2	AST0, ABT0

3-Thiopheneethanol	2	AST0, ABT0
3-Vinyl-1 2-dithiacyclohex-4-ene	2	AST0, ABT0
3H-1 2 4-Triazole-3-thione 2 4-dihydro-4-methyl-	2	AST0, ABT0
4-Cyclopentene-1 3-dione	2	ABT12, AST12
4H-Pyran-4-one 2 3-dihydro-3 5-dihydroxy-6-methyl-	2	ABT12, AST12
5-(2-Thienyl)pentanoic acid	2	AST0, ABT0
5-Hydroxymethylfurfural	2	ABT12, AST12
Acetic acid	2	ABT12, AST12
Amidinothiourea	2	AST0, ABT0
Benzene 1 3-bis(1 1-dimethylethyl)-	2	ABT12, AST12
Butyrolactone	2	ABT12, AST12
Ethanone 1-(1H-pyrrol-2-yl)-	2	ABT12, AST12
Ethanone 1-(2-furanyl)-	2	ABT12, AST12
Formic acid	2	ABT12, AST12
Furfural	2	ABT12, AST12
Hexadecane	2	AST0, ABT0
Hydrocoumarin	2	AST0, ABT0
Methyl 2-furoate	2	ABT12, AST12
Nicotinyl alcohol	2	ABT12, AST12
Phenol	2	ABT12, AST12
Thiazole 5-methoxy-	2	AST0, ABT0
**AB**		
1 3 5-Trithiane	3	ABT0, ABT6, ABT12
1 3-Dithiane	3	ABT0, ABT6, ABT12
2-Pentanol 4-methyl-	3	ABT0, ABT6, ABT12
3-Vinyl-1 2-dithiacyclohex-5-ene	3	ABT0, ABT6, ABT12
Butane 1-isothiocyanato-	3	ABT0, ABT6, ABT12
Butylated Hydroxytoluene	3	ABT0, ABT6, ABT12
Cyclohexasiloxane dodecamethyl-	3	ABT0, ABT6, ABT12
Diallyl disulphide	3	ABT0, ABT6, ABT12
Diallyl sulfide	3	ABT0, ABT6, ABT12
Dodecane	3	ABT0, ABT6, ABT12
Dodecanoic acid	3	ABT0, ABT6, ABT12
Indole	3	ABT0, ABT6, ABT12
Phenol 2 4-bis(1 1-dimethylethyl)-	3	ABT0, ABT6, ABT12
Phenol 4-(1 1 3 3-tetramethylbutyl)-	3	ABT0, ABT6, ABT12
Tetradecane	3	ABT0, ABT6, ABT12
Trisulfide di-2-propenyl	3	ABT0, ABT6, ABT12
1 2-Dithiolane	2	ABT0, ABT6
1 3-Dithiane 2-methyl-	2	ABT0, ABT6
1 4-Dithiane	2	ABT0, ABT6
1-Propanamine N 2-dimethyl-	2	ABT0, ABT6
1H-Pyrrole-2-carboxaldehyde	2	ABT6, ABT12
2 4-Dimethyl-5 6-dithia-2 7-nonadienal	2	ABT0, ABT6
2 4-Dithiapentane	2	ABT0, ABT6
2 5-Thiophenedicarboxaldehyde	2	ABT6, ABT12
2-Furancarboxaldehyde 5-methyl-	2	ABT6, ABT12
2-Furanmethanol	2	ABT6, ABT12
2-Propanone 1-hydroxy-	2	ABT6, ABT12
2-Thiazolamine 5-nitro-	2	ABT0, ABT6
4-Cyclopentene-1 3-dione	2	ABT6, ABT12
4-Pyridinol	2	ABT6, ABT12
4-Thiazolidinone 3-amino-2-thioxo-	2	ABT0, ABT6
4H-Pyran-4-one 2 3-dihydro-3 5-dihydroxy-6-methyl-	2	ABT6, ABT12
5-(2-Thienyl)pentanoic acid	2	ABT0, ABT6
5-Hydroxymethylfurfural	2	ABT6, ABT12

Acetic acid	2	ABT6, ABT12
Acetylpyrazine	2	ABT6, ABT12
Amidinothiourea	2	ABT0, ABT6
Benzene 1 3-bis(1 1-dimethylethyl)-	2	ABT6, ABT12
Carbon dioxide	2	ABT0, ABT12
Dehydromevalonic lactone	2	ABT6, ABT12
Dimethyl trisulfide	2	ABT6, ABT12
Ethanone 1-(1H-pyrrol-2-yl)-	2	ABT6, ABT12
Ethanone 1-(2-furanyl)-	2	ABT6, ABT12
Furfural	2	ABT6, ABT12
Hexadecane	2	ABT0, ABT6
Hexanoic acid	2	ABT6, ABT12
Naphthalene-2 6-dicarboxylic acid bis-(4-nonyl-phenyl ester	2	ABT0, ABT6
Pentasiloxane dodecamethyl-	2	ABT0, ABT6
Phenol	2	ABT6, ABT12
Pyrazine 2 6-dimethyl-	2	ABT6, ABT12
Thiazole 5-methoxy-	2	ABT0, ABT6
**AS**		
1 3-Dithiane	3	AST0, AST6, AST12
2 4-Dithiapentane	3	AST0, AST6, AST12
3-Vinyl-1 2-dithiacyclohex-5-ene	3	AST0, AST6, AST12
4-Thiazolidinone 3-amino-2-thioxo-	3	AST0, AST6, AST12
Butylated Hydroxytoluene	3	AST0, AST6, AST12
Diallyl disulphide	3	AST0, AST6, AST12
Diallyl sulfide	3	AST0, AST6, AST12
Dimethyl trisulfide	3	AST0, AST6, AST12
Dodecanoic acid	3	AST0, AST6, AST12
Indole	3	AST0, AST6, AST12
Phenol 2 4-bis(1 1-dimethylethyl)-	3	AST0, AST6, AST12
Propanoic acid	3	AST0, AST6, AST12
Tetradecane	3	AST0, AST6, AST12
Trisulfide di-2-propenyl	3	AST0, AST6, AST12
1 2-Cyclopentanedione 3-methyl-	2	AST6, AST12
1 3 5-Trithiane	2	AST0, AST6
1 3-Benzenedithiol	2	AST0, AST6
1 3-Dithiane 2 2-dimethyl-	2	AST6, AST12
1 3-Dithiane 2-methyl-	2	AST0, AST6
1H-Imidazole-4-carboxylic acid methyl ester	2	AST6, AST12
1H-Pyrrole-2-carboxaldehyde	2	AST6, AST12
2 5-Furandicarboxaldehyde	2	AST6, AST12
2 5-Thiophenedicarboxaldehyde	2	AST6, AST12
2(5H)-Furanone	2	AST6, AST12
2-Furancarboxaldehyde 5-methyl-	2	AST6, AST12
2-Furancarboxylic acid hydrazide	2	AST6, AST12
2-Furanmethanol	2	AST6, AST12
2-Propanone 1-hydroxy-	2	AST6, AST12
2-Thiazolamine 5-nitro-	2	AST0, AST6
3-Vinyl-1 2-dithiacyclohex-4-ene	2	AST0, AST6
3H-1 2 4-Triazole-3-thione 2 4-dihydro-4-methyl-	2	AST0, AST6
4-Cyclopentene-1 3-dione	2	AST6, AST12
4H-Pyran-4-one 2 3-dihydro-3 5-dihydroxy-6-methyl-	2	AST6, AST12
5-(2-Thienyl)pentanoic acid	2	AST0, AST6
5-Hydroxymethylfurfural	2	AST6, AST12
Acetic acid	2	AST6, AST12
Amidinothiourea	2	AST0, AST6
Butane 1-isothiocyanato-	2	AST6, AST12

Carbon dioxide	2	AST6, AST12
Cyclohexasiloxane dodecamethyl-	2	AST6, AST12
Dodecane	2	AST6, AST12
Ethanone 1-(2-furanyl)-	2	AST6, AST12
Formic acid	2	AST6, AST12
Furfural	2	AST6, AST12
Methyl 2-furoate	2	AST6, AST12
Phenol	2	AST6, AST12
Silane ethoxytrimethyl-	2	AST0, AST6
Sulfide allyl methyl	2	AST0, AST6
Thiophene 3 4-dimethyl-	2	AST0, AST6

Supplemental references:

45 Walter, J., Hertel, C., Tannock, G. W., Lis, C. M., Munro, K., & Hammes, W. P. (2001). Detection of Lactobacillus, Pediococcus, Leuconostoc, and Weissella species in human feces by using group-specific PCR primers and denaturing gradient gel electrophoresis. Applied and environmental microbiology, 67(6), 2578-2585.

46 Masco, L., Ventura, M., Zink, R., Huys, G., & Swings, J. (2004). Polyphasic taxonomic analysis of Bifidobacterium animalis and Bifidobacterium lactis reveals relatedness at the subspecies level: reclassification of Bifidobacterium animalis as Bifidobacterium animalis subsp. animalis subsp. nov. and Bifidobacterium lactis as Bifidobacterium animalis subsp. lactis subsp. nov. International Journal of Systematic and Evolutionary Microbiology, 54(4), 1137-1143.

47 Zhou, P., Bogan, J. A., Welch, K., Pickett, S. R., Wang, H. J., Zaritsky, A., & Helmstetter, C. E. (1997). Gene transcription and chromosome replication in Escherichia coli. Journal of bacteriology, 179(1), 163-169.
